# The genome sequence of the bramble shoot moth, *Notocelia uddmanniana* (Linnaeus, 1758)

**DOI:** 10.12688/wellcomeopenres.17488.1

**Published:** 2021-12-15

**Authors:** Douglas Boyes, Peter W.H. Holland, University of Oxford and Wytham Woods Genome Acquisition Lab, Darwin Tree of Life Barcoding collective, Wellcome Sanger Institute Tree of Life programme, Wellcome Sanger Institute Scientific Operations: DNA Pipelines collective, Tree of Life Core Informatics collective, Darwin Tree of Life Consortium

**Affiliations:** 1 UK Centre for Ecology and Hydrology, Wallingford, Oxfordshire, UK; 2 Department of Zoology, University of Oxford, Oxford, UK

**Keywords:** Notocelia uddmanniana, bramble shoot moth, genome sequence, chromosomal, Lepidoptera

## Abstract

We present a genome assembly from an individual male *Notocelia uddmanniana* (the bramble shoot moth; Arthropoda; Insecta; Lepidoptera; Tortricidae). The genome sequence is 794 megabases in span. The majority of the assembly, 99.96%, is scaffolded into 28 chromosomal pseudomolecules, with the Z sex chromosome assembled.

## Species taxonomy

Eukaryota; Metazoa; Ecdysozoa; Arthropoda; Hexapoda; Insecta; Pterygota; Neoptera; Endopterygota; Lepidoptera; Glossata; Ditrysia; Tortricoidea; Tortricidae; Olethreutinae; Eucosmini; Notocelia; *Notocelia uddmanniana* (Linnaeus, 1758) (NCBI:txid1594315).

## Background

*Notocelia uddmanniana* (bramble shoot moth) is widely distributed across Western Europe and North Africa, with records further east from Kazakhstan to China. The larvae feed on brambles ( *Rubus* sp.), occurring commonly where these species exist, and occasionally cause damage to cultivated varieties ( Gordon *et al.*, 1997). Eggs are laid singly on the foodplant, where larvae feed within a folded leaf and later within the tips of growing shoots; larvae overwinter in a silken web on the foodplant stem before recommencing feeding in spring ( Dicker, 1939). *Notocelia uddmanniana* also occupies woodland, and is distributed widely throughout the UK, occurring more commonly in the south. The genome of *N. uddmanniana* was sequenced as part of the Darwin Tree of Life Project, a collaborative effort to sequence all of the named eukaryotic species in the Atlantic Archipelago of Britain and Ireland. Here we present a chromosomally complete genome sequence for *N. uddmanniana*, based on one male specimen from Wytham Woods, Oxfordshire, UK.

## Genome sequence report

**Figure 1.**
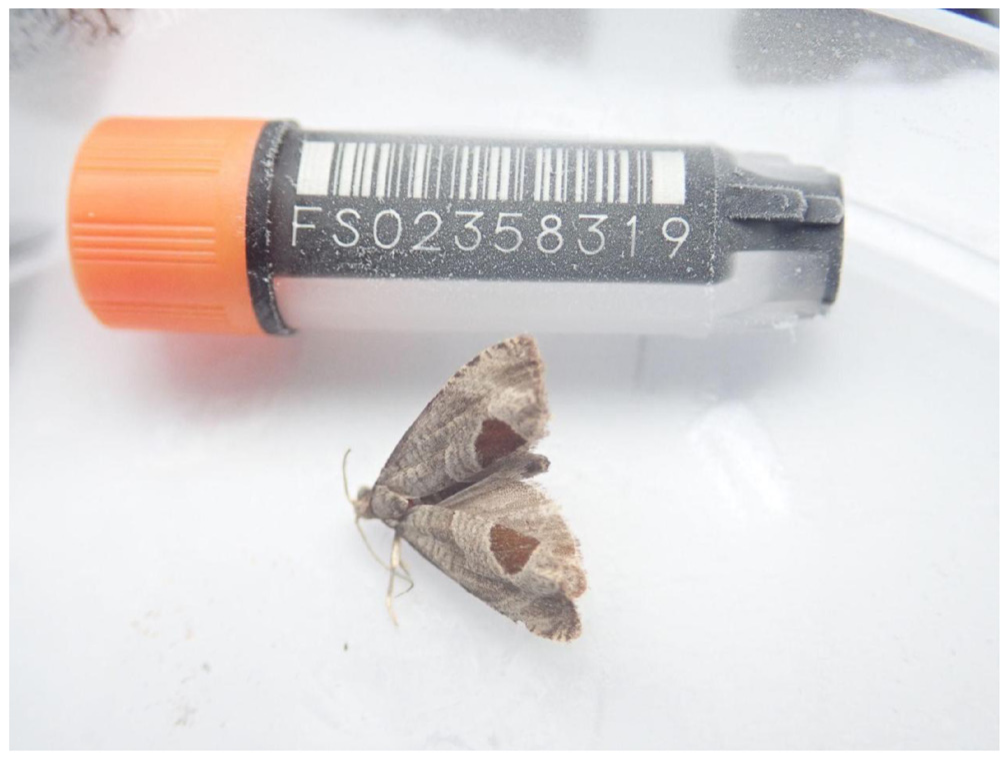
Image of the ilNotUddm1 specimen taken during preservation and processing.

The genome was sequenced from a single male *N. uddmanniana* ( Figure 1) collected from Wytham Woods, Oxfordshire, UK (latitude 51.772, longitude -1.338). A total of 18-fold coverage in Pacific Biosciences single-molecule long reads (N50 16 kb) and 49-fold coverage in 10X Genomics read clouds were generated. Primary assembly contigs were scaffolded with chromosome conformation Hi-C data. Manual assembly curation corrected 165 missing/misjoins and removed 72 haplotypic duplications, reducing the assembly length by 2.16% and the scaffold number by 64.71%, and increasing the scaffold N50 by 9.53%.

**Figure 2.**
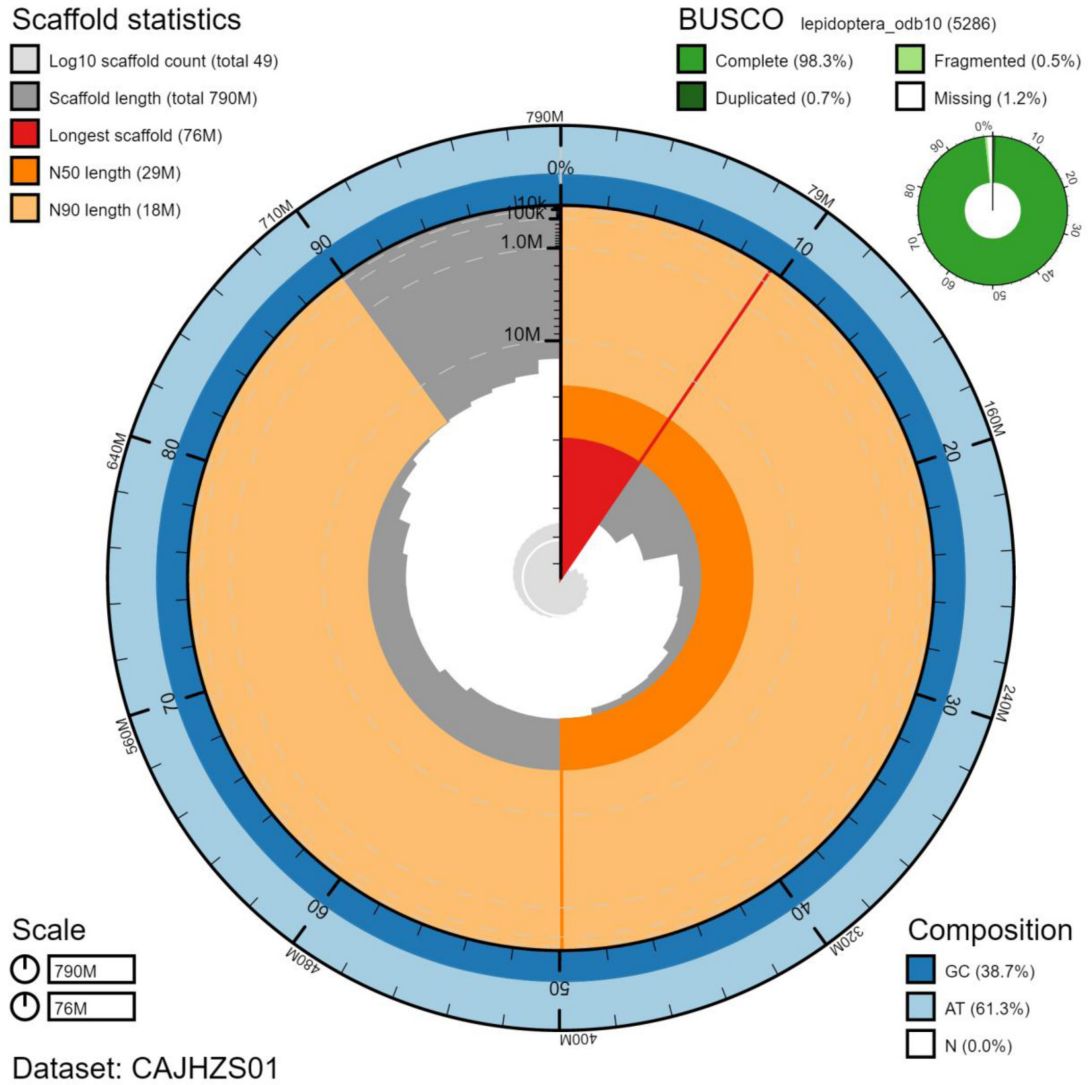
Genome assembly of *Notocelia uddmanniana*, ilNotUddm1.1: metrics. The BlobToolKit Snailplot shows N50 metrics and BUSCO gene completeness. The main plot is divided into 1,000 size-ordered bins around the circumference with each bin representing 0.1% of the 794,123,667 bp assembly. The distribution of chromosome lengths is shown in dark grey with the plot radius scaled to the longest chromosome present in the assembly (75,621,453 bp, shown in red). Orange and pale-orange arcs show the N50 and N90 chromosome lengths (28,990,537 and 17,668,102 bp), respectively. The pale grey spiral shows the cumulative chromosome count on a log scale with white scale lines showing successive orders of magnitude. The blue and pale-blue area around the outside of the plot shows the distribution of GC, AT and N percentages in the same bins as the inner plot. A summary of complete, fragmented, duplicated and missing BUSCO genes in the lepidoptera_odb10 set is shown in the top right. An interactive version of this figure is available at https://blobtoolkit.genomehubs.org/view/ilNotUddm1.1/dataset/CAJHZS01/snail.

The final assembly has a total length of 794 Mb in 49 sequence scaffolds with a scaffold N50 of 29 Mb ( Table 1). Of the assembly sequence, 99.96% was assigned to 28 chromosomal-level scaffolds, representing 27 autosomes (numbered by sequence length), and the Z sex chromosome ( Figure 2– Figure 5; Table 2). The assembly has a BUSCO ( Simão *et al.*, 2015) completeness of 98.9% using the lepidoptera_odb10 reference set. While not fully phased, the assembly deposited is of one haplotype. Contigs corresponding to the second haplotype have also been deposited.

**Table 1.** Genome data for *Notocelia uddmanniana*, ilNotUddm1.1.

*Project accession data*	
Assembly identifier	ilNotUddm1
Species	*Notocelia uddmanniana*
Specimen	ilNotUddm1
NCBI taxonomy ID	NCBI:txid1594315
BioProject	PRJEB42137
BioSample ID	SAMEA7519916
Isolate information	Male, whole organism
*Raw data accessions*	
PacificBiosciences SEQUEL II	ERR6590584
10X Genomics Illumina	ERR6002710-ERR6002713
Hi-C Illumina	ERR6002707-ERR6002709
*Genome assembly*	
Assembly accession	GCA_905163555.1
*Accession of alternate haplotype*	GCA_905163575.1
Span (Mb)	794
Number of contigs	238
Contig N50 length (Mb)	7
Number of scaffolds	49
Scaffold N50 length (Mb)	29
Longest scaffold (Mb)	51
BUSCO * genome score	C:98.3%[S:97.6%,D:0.7%], F:0.5%,M:1.2%,n:5286

*BUSCO scores based on the lepidoptera_odb10 BUSCO set using v5.1.2. C= complete [S= single copy, D=duplicated], F=fragmented, M=missing, n=number of orthologues in comparison. A full set of BUSCO scores is available at https://blobtoolkit.genomehubs.org/view/ilNotUddm1.1/dataset/CAJHZS01/busco.

**Figure 3.**
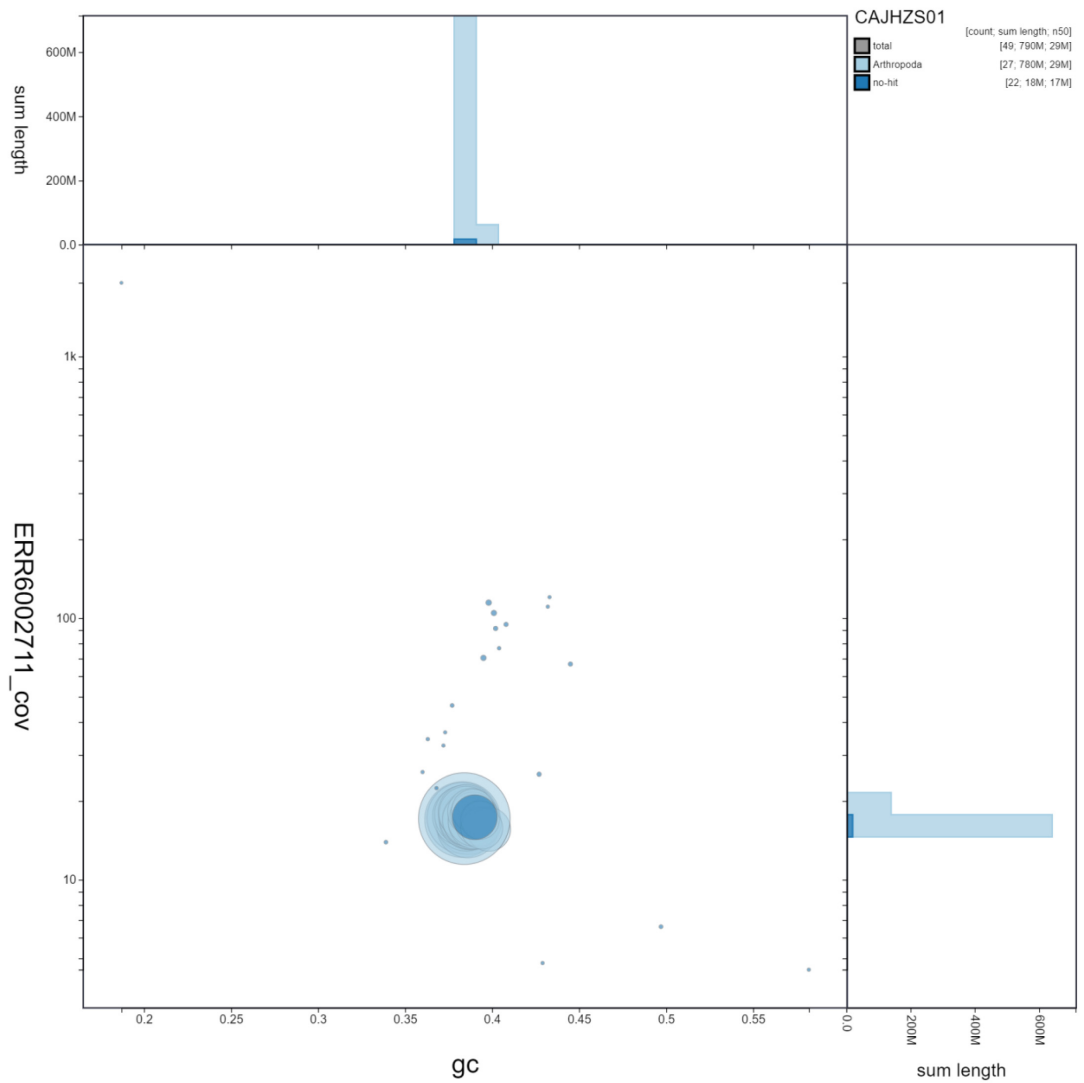
Genome assembly of *Notocelia uddmanniana*, ilNotUddm1.1: GC coverage. BlobToolKit GC-coverage plot. Scaffolds are coloured by phylum. Circles are sized in proportion to scaffold length. Histograms show the distribution of scaffold length sum along each axis. An interactive version of this figure is available at https://blobtoolkit.genomehubs.org/view/ilNotUddm1.1/dataset/CAJHZS01/blob.

**Figure 4.**
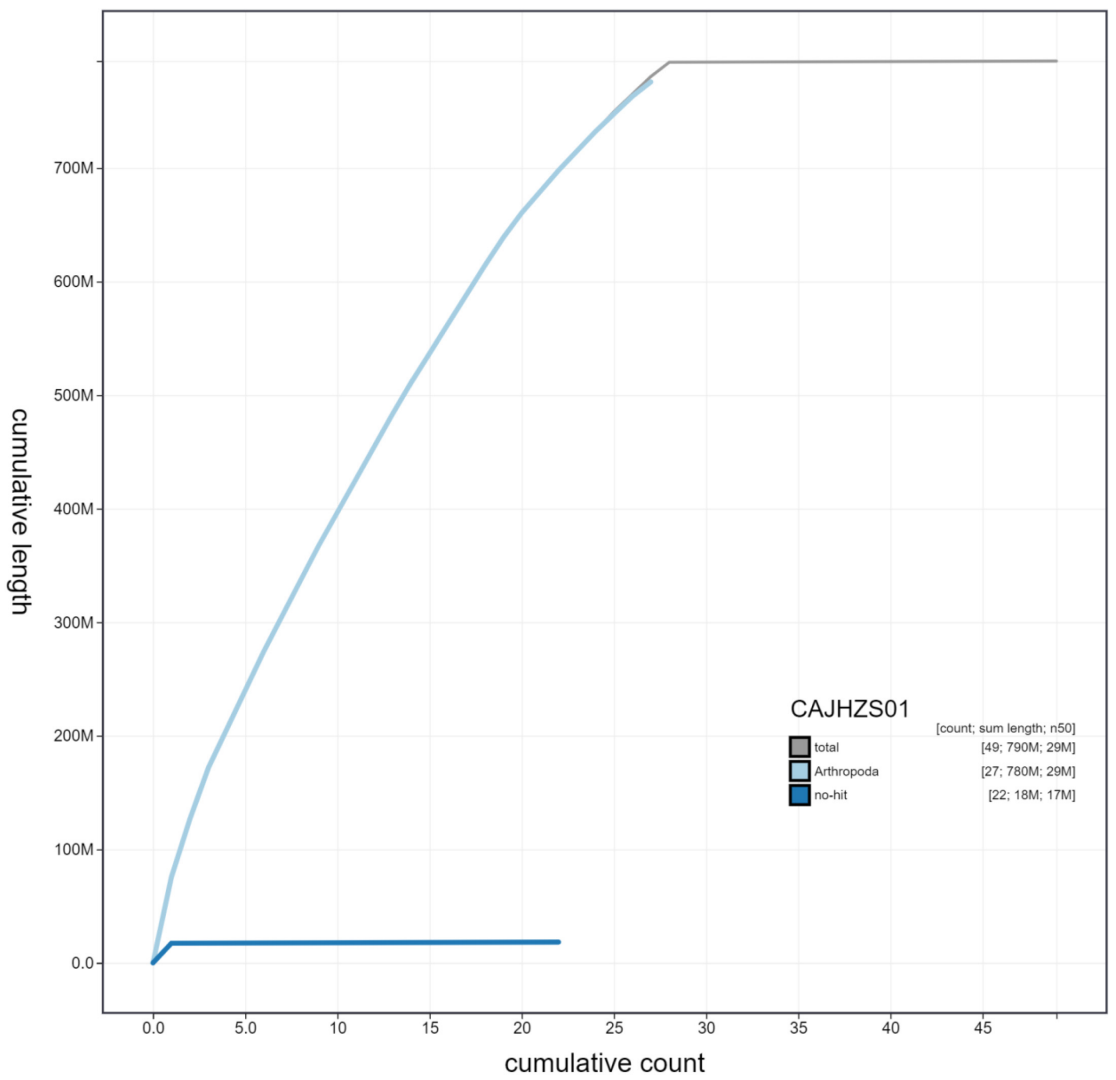
Genome assembly of *Notocelia uddmanniana*, ilNotUddm1.1: cumulative sequence. BlobToolKit cumulative sequence plot. The grey line shows cumulative length for all scaffolds. Coloured lines show cumulative lengths of scaffolds assigned to each phylum using the buscogenes taxrule. An interactive version of this figure is available at https://blobtoolkit.genomehubs.org/view/ilNotUddm1.1/dataset/CAJHZS01/cumulative.

**Figure 5.**
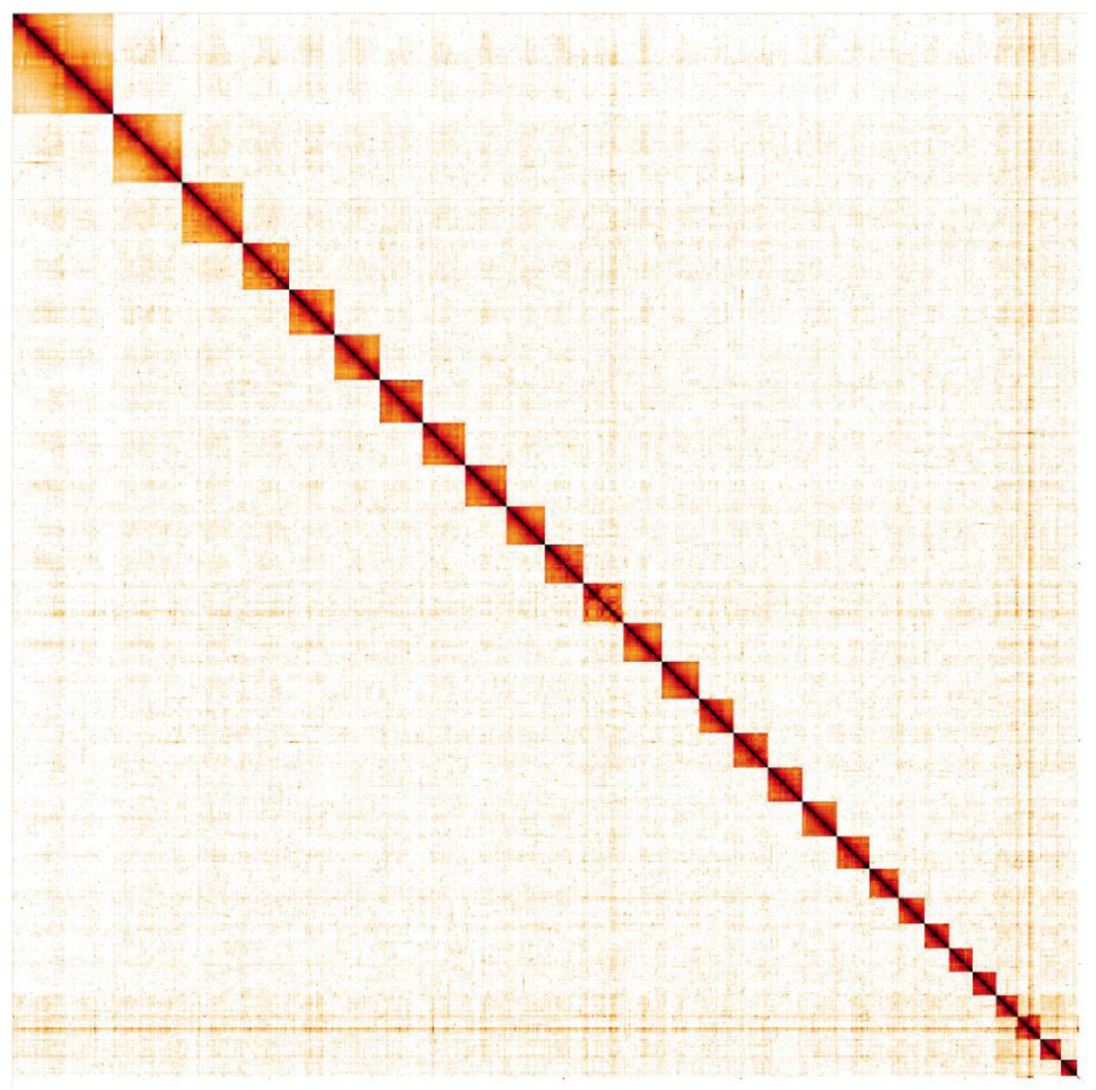
Genome assembly of *Notocelia uddmanniana*, ilNotUddm1.1: Hi-C contact map. Hi-C contact map of the ilNotUddm1.1 assembly, visualised in HiGlass. Chromosomes are given in order of size from left to right and top to bottom.

**Table 2.** Chromosomal pseudomolecules in the genome assembly of *Notocelia uddmanniana*, ilNotUddm1.1.

INSDC accession	Chromosome	Size (Mb)	GC%
LR991053.1	1	51.12	38.3
LR991054.1	2	44.98	38.3
LR991055.1	3	34.90	38.6
LR991056.1	4	33.91	38.6
LR991057.1	5	33.55	38.5
LR991058.1	6	31.63	38.3
LR991059.1	7	31.24	38.4
LR991060.1	8	30.81	38.7
LR991061.1	9	29.12	38.8
LR991062.1	10	28.99	38.6
LR991063.1	11	28.96	38.8
LR991064.1	12	28.81	38.6
LR991065.1	13	27.46	38.5
LR991066.1	14	25.91	38.7
LR991067.1	15	25.88	38.7
LR991068.1	16	25.71	38.5
LR991069.1	17	25.63	38.7
LR991070.1	18	24.47	39
LR991071.1	19	21.77	38.9
LR991072.1	20	19.36	38.8
LR991073.1	21	18.18	39.2
LR991074.1	22	17.67	38.9
LR991075.1	23	17.30	39
LR991077.1	24	15.87	38.9
LR991076.1	25	16.51	39.8
LR991078.1	26	15.07	39.4
LR991079.1	27	12.65	39.3
LR991052.1	Z	75.62	38.4
LR991080.1	MT	0.02	18.8
-	Unplaced	1.01	40.8

## Methods

### Sample acquisition, DNA extraction and sequencing

A single male *M. uddmanniana* (ilNotUddm1) was collected from Wytham Woods, Oxfordshire, UK (latitude 51.772, longitude -1.338) by Douglas Boyes, UKCEH, using a light trap. The specimen was identified by the same individual and preserved on dry ice.

DNA was extracted from whole organism tissue at the Wellcome Sanger Institute (WSI) Scientific Operations core from the whole organism using the Qiagen MagAttract HMW DNA kit, according to the manufacturer’s instructions. Pacific Biosciences HiFi circular consensus and 10X Genomics read cloud sequencing libraries were constructed according to the manufacturers’ instructions. Sequencing was performed by the Scientific Operations core at the Wellcome Sanger Institute on Pacific Biosciences SEQUEL II and Illumina HiSeq X instruments. Hi-C data were generated from remaining whole organism tissue using the Arima v1.0 kit and sequenced on HiSeq X.

### Genome assembly

Assembly was carried out with Hifiasm ( Cheng *et al.*, 2021); haplotypic duplication was identified and removed with purge_dups ( Guan *et al.*, 2020), without the -e flag. One round of polishing was performed by aligning 10X Genomics read data to the assembly with longranger align, calling variants with freebayes ( Garrison & Marth, 2012). The assembly was then scaffolded with Hi-C data ( Rao *et al.*, 2014) using SALSA2 ( Ghurye *et al.*, 2019). The assembly was checked for contamination and corrected using the gEVAL system ( Chow *et al.*, 2016) as described previously ( Howe *et al.*, 2021). Manual curation ( Howe *et al.*, 2021) was performed using gEVAL, HiGlass ( Kerpedjiev *et al.*, 2018) and Pretext. The mitochondrial genome was assembled using MitoHiFi ( Uliano-Silva *et al.*, 2021) and annotated using MitoFinder ( Allio *et al.*, 2020). The genome was analysed and BUSCO scores generated within the BlobToolKit environment ( Challis *et al.*, 2020). Table 3 contains a list of all software tool versions used, where appropriate.

**Table 3.** Software tools used.

Software tool	Version	Source
Hifiasm	0.12	Cheng *et al.*, 2021
purge_dups	1.2.3	Guan *et al.*, 2020
SALSA2	2.2	Ghurye *et al.*, 2019
longranger align	2.2.2	https://support.10xgenomics.com/genome-exome/software/ pipelines/latest/advanced/other-pipelines
freebayes	1.3.1-17-gaa2ace8	Garrison & Marth, 2012
MitoHiFi	1	https://github.com/marcelauliano/MitoHiFi
gEVAL	N/A	Chow *et al.*, 2016
HiGlass	1.11.6	Kerpedjiev *et al.*, 2018
PretextView	0.1.x	https://github.com/wtsi-hpag/PretextView
BlobToolKit	2.6.2	Challis *et al.*, 2020

### Ethics/compliance issues

The materials that have contributed to this genome note have been supplied by a Darwin Tree of Life Partner. The submission of materials by a Darwin Tree of Life Partner is subject to the Darwin Tree of Life Project Sampling Code of Practice. By agreeing with and signing up to the Sampling Code of Practice, the Darwin Tree of Life Partner agrees they will meet the legal and ethical requirements and standards set out within this document in respect of all samples acquired for, and supplied to, the Darwin Tree of Life Project. Each transfer of samples is further undertaken according to a Research Collaboration Agreement or Material Transfer Agreement entered into by the Darwin Tree of Life Partner, Genome Research Limited (operating as the Wellcome Sanger Institute), and in some circumstances other Darwin Tree of Life collaborators.

## Data availability

European Nucleotide Archive: Notocelia uddmanniana (bramble shoot). Accession number PRJEB42137: https://www.ebi.ac.uk/ena/browser/view/PRJEB42037.

The genome sequence is released openly for reuse. The *N. uddmanniana* genome sequencing initiative is part of the Darwin Tree of Life (DToL) project. All raw sequence data and the assembly have been deposited in INSDC databases. The genome will be annotated and presented through the Ensembl pipeline at the European Bioinformatics Institute. Raw data and assembly accession identifiers are reported in Table 1.

## Author information

Members of the University of Oxford and Wytham Woods Genome Acquisition Lab are listed here: https://doi.org/10.5281/zenodo.5746938.

Members of the Darwin Tree of Life Barcoding collective are listed here: https://doi.org/10.5281/zenodo.5744972.

Members of the Wellcome Sanger Institute Tree of Life programme are listed here: https://doi.org/10.5281/zenodo.5744840.

Members of Wellcome Sanger Institute Scientific Operations: DNA Pipelines collective are listed here: https://doi.org/10.5281/zenodo.5746904.

Members of the Tree of Life Core Informatics collective are listed here: https://doi.org/10.5281/zenodo.5743293.

Members of the Darwin Tree of Life Consortium are listed here: https://doi.org/10.5281/zenodo.5638618.
