# Supplementary material for: The genome sequence of the bramble shoot moth, Notocelia uddmanniana (Linnaeus, 1758)
Source: Wellcome Open Res. 2021 Dec 15;6:348. [Version 1] doi: 10.12688/wellcomeopenres.17488.1 (PMC11339589; doi:10.12688/wellcomeopenres.17488.1)
Supplement: Peer review history

# Reviewer report: Reviewer response for version 1

**DOI:** 10.21956/wellcomeopenres.19338.r54332

**Published:** 2024-08-21

**Authors:** Hidemasa Bono

**Affiliations:** 1 Hiroshima University, Kagamiyam, Japan

Authors described genome sequencing of the bramble shoot moth, *Notocelia uddmanniana. *There are no other assemblies in the INSDC records besides the one submitted by the author. There are no problem in sample acquisition and DNA extraction. Furthermore, the data analysis method described in the manuscript is modern and appropriate for the genome assembly of insect species.

 There is a typo in the Data availability section; PRJEB42137 should be PRJEB42 **0**37. It should be fixed before the final indexing because this information is very critical for 'Data Note' manuscript.

Are sufficient details of methods and materials provided to allow replication by others?

Yes

Is the rationale for creating the dataset(s) clearly described?

Yes

Are the datasets clearly presented in a useable and accessible format?

Yes

Are the protocols appropriate and is the work technically sound?

Yes

Reviewer Expertise:

Genome biology

I confirm that I have read this submission and believe that I have an appropriate level of expertise to confirm that it is of an acceptable scientific standard.

# Reviewer report: Reviewer response for version 1

**DOI:** 10.21956/wellcomeopenres.19338.r76909

**Published:** 2024-04-17

**Authors:** Violaine Llaurens

**Affiliations:** 1 CNRS, Paris, France

The article accurately describes the chromosome-level assembly of the genome of a male *Notocelia uddmanniana *sampled in Oxfordshire. These new data open new research avenue in different scientific fields from ecology to evolution in Lepidoptera. A single male was sequenced, and the assembly show that the genome size is 794 mb long, and is arranged in 28 different chromosomes. At the moment, this genome remains to be annotated.

 I have only a minor comment on this genome release : It is unclear to me whether the chromosome 1, which is the largest chromosome, corresponds to the Z-chromosome ?

Are sufficient details of methods and materials provided to allow replication by others?

Yes

Is the rationale for creating the dataset(s) clearly described?

Yes

Are the datasets clearly presented in a useable and accessible format?

Yes

Are the protocols appropriate and is the work technically sound?

Yes

Reviewer Expertise:

Evolutionary biology

I confirm that I have read this submission and believe that I have an appropriate level of expertise to confirm that it is of an acceptable scientific standard.

# Reviewer report: Reviewer response for version 1

**DOI:** 10.21956/wellcomeopenres.19338.r79177

**Published:** 2024-04-15

**Authors:** Jeffrey Marcus

**Affiliations:** 1 University of Manitoba, Winnipeg, Manitoba, Canada

In this manuscript, the authors describe the sequencing and assembly of the *Notocelia uddmanniana *genome using DNA from a single male specimen collected in the UK.  The primary genome sequence assembly includes proposed chromosomal pseudomolecule sequences for 27 autosomes, the Z sex chromosome, and a complete mitochondrial genome. No females were collected, so the W sex chromosome was not sequenced *.* Overall, this is a solid contribution to the scientific literature.

 Some suggestions to the authors:

1. Methods:  The date of collection for the sequenced *Notocelia uddmanniana* specimen is not included in the manuscript. It is best practice to include collection dates for all wild-caught insect specimens used for genome sequencing because it is not unusual for cryptic species pairs to be distinguishable by differences in phenology of adult emergence. If it should come to pass that genus *Notocelia* includes cryptic species, knowing the specimen collection date can help future researchers determine to which taxon this sequenced genome should be assigned, especially since it appears that the entire specimen was used for DNA extraction and is no longer available for morphological examination.
2. For future work, I suggest that the researchers preferentially sequence the heterogametic sex when assembling genomes for previously unstudied species (in the case of Lepidoptera, the heterogametic sex is female), so that draft assembles can be prepared for both sex chromosomes.

Are sufficient details of methods and materials provided to allow replication by others?

No

Is the rationale for creating the dataset(s) clearly described?

Yes

Are the datasets clearly presented in a useable and accessible format?

Yes

Are the protocols appropriate and is the work technically sound?

Yes

Reviewer Expertise:

Evolutionary biology of insects, phylogenomics

I confirm that I have read this submission and believe that I have an appropriate level of expertise to confirm that it is of an acceptable scientific standard, however I have significant reservations, as outlined above.
